# Comparison of Local Recurrence After Simple and Skin-Sparing Mastectomy Performed in Patients with Ductal Carcinoma In Situ

**DOI:** 10.1245/s10434-016-5673-6

**Published:** 2016-11-11

**Authors:** Simon Timbrell, Sarah Al-Himdani, Oliver Shaw, Kian Tan, Julie Morris, Nigel Bundred

**Affiliations:** Academic Surgery, University Hospital of South Manchester, Manchester, UK

## Abstract

**Background:**

The incidence of ductal carcinoma in situ (DCIS) is increasing with the use of screening mammography, and approximately 30% of all women diagnosed with DCIS are treated by mastectomy. There is increasing use of a skin-sparing mastectomy (SSM) approach to surgically excise DCIS as this facilitates immediate breast reconstruction. The rates of locoregional recurrence (LRR) after simple mastectomy performed for pure DCIS are historically reported as 1%; however, international data suggest that LRR after SSM may be higher.

**Methods:**

To determine our rates of LRR and compare the effect of the type of mastectomy performed, we undertook a retrospective review of all patients who underwent a mastectomy for pure DCIS at our institution between 2000 and 2010.

**Results:**

In total, 199 patients underwent a mastectomy for pure DCIS (with eight local recurrences), all of which were invasive ductal carcinoma. The recurrences all occurred after SSM, which was associated with a higher 5-year LRR of 5.9% (5/102) compared with 0% in the simple mastectomy group (0/97; *p* *=* 0.012), log-rank. Univariate analysis showed the two factors that predicted the risk of recurrence were a young age at mastectomy and close or involved margins.

**Conclusions:**

These data highlight the importance of achieving clear margins, especially in young women with estrogen receptor-negative DCIS who have a higher risk of invasive recurrence. Women undergoing a mastectomy for DCIS should be counseled as to the importance of achieving clear margins and the potential increased need for further excision, post-mastectomy radiotherapy and post-reconstruction mammography in order to prevent LRR after SSM.

**Electronic supplementary material:**

The online version of this article (doi:10.1245/s10434-016-5673-6) contains supplementary material, which is available to authorized users.

Ductal carcinoma in situ (DCIS) is a pre-invasive form of breast cancer where malignant cells are confined within the ductal basement membrane.^1^ Its incidence has increased with the introduction of screening mammography and it accounts for 21% of screen-detected malignancies in the UK.^2^ Surgical excision involves breast-conserving surgery in the form of a wide local excision (WLE) or removal of the entire breast by mastectomy. Mastectomy is indicated where there is extensive DCIS to breast size which does not allow for a cosmetically or surgically acceptable WLE, or in the presence of multifocal disease.^1^ According to *The Second All Breast Cancer Report*, 38% of non-invasive breast cancers were treated by mastectomy in 2007.^3^

A simple mastectomy was traditionally performed whereby the entire breast is removed with a large ellipse of overlying skin. Increasing use of skin-sparing mastectomy (SSM) facilitates immediate breast reconstruction by preserving the patients’ skin coverage with improved aesthetic and psychological outcome.^4^,^5^ SSM involves excision of the breast via a smaller elliptical incision, resulting in less scarring and fewer surgical procedures.^4^

The larger surface area of SSM skin flaps increases the potential to leave residual breast tissue. Achieving the ideal mastectomy flap that is thin enough to remove all breast tissue but thick enough to keep subdermal vessels and support an adequate blood supply is difficult.^6^ Histological studies have shown that this plane is absent in 44% of cases and its thickness varies from 0 to 29 mm, with a median of 10 mm.^7^,^8^ A mastectomy flap of 4–5 mm led to flap necrosis rates of 17%, whereas others reported less than 5% with flaps thicker than 5 mm.^9^,^10^ Higher rates of locoregional recurrence (LRR) after SSM were initially reported for invasive cancer^11^ but were not confirmed in a subsequent meta-analysis of LRR (4% in simple mastectomy vs. 6.2% in SSM).^12^ This analysis encompassed all forms of breast cancer, with no subgroup analysis in DCIS.^12^

LRR after mastectomy for DCIS has historically been demonstrated as low, with the UK SLOANE audit reporting a 1% LRR,^13^ and a meta-analysis incorporating 1574 patients demonstrating an LRR of 1.4%.^14^ DCIS is associated with less clinically apparent disease, making identification of lesions intraoperatively more difficult.^1^ There is often more widespread multi-focal disease with a greater chance of a separate DCIS foci away from the primary lesion than in invasive ductal carcinoma (IDC).^15^ This emphasizes the importance of removing all breast tissue during a mastectomy as residual parenchyma may contain another focus of DCIS.^5^,^15^ Cao et al. removed an additional superficial margin directly over the tumor in 168 patients. Thirty-eight percent had a positive superficial specimen margin, 13 (20%) of whom had residual carcinoma in the additional biopsy. Factors associated with a positive flap biopsy were the presence of extensive DCIS and a thicker superficial flap biopsy.^16^ In 2007, 27% of patients undergoing a mastectomy for DCIS had an SSM with immediate breast reconstruction compared with 10% in invasive disease.^3^ The higher rates of SSM use in DCIS are to be expected as these patients are unlikely to require adjuvant radiotherapy.^13^ Despite SSMs increasing use in DCIS, there are little data on oncological outcomes in simple mastectomy compared with SSM. Emerging data from the US highlight that LRR after SSM for DCIS is high, at 5%.^17^ Higher LRR has been reported in the UK, with a 15-year retrospective review of screen-detected lesions in the West Midlands demonstrating a 3.1% 5-year LRR and an 8% 15-year LRR.^18^

We aimed to determine our LRR after mastectomy for DCIS, comparing SSMs and simple mastectomies and evaluating which factors predicted LRR.

## Patients

We undertook a retrospective review of all patients who had a mastectomy for pure DCIS at the University Hospital of South Manchester between 2000 and 2010, after hospital ethical approval was obtained.

The operation notes were reviewed to collect data on the type of mastectomy, the reconstructive strategy used, and the type of axillary surgery.

Pathological reports were reviewed and data collected on histological type, grade, size of DCIS, and margin status. The presence or absence of microinvasion, lymphovascular invasion, and comedonecrosis was recorded, and the available molecular phenotype information, including estrogen receptor (ER) status, progesterone receptor (PR) status, and human epidermal growth factor receptor 2 (HER2) status, for those patients recruited to clinical trials was also recorded.

Clinical notes were reviewed to evaluate the method of presentation, use of adjuvant therapy, and follow-up data. When recurrences did occur, we gathered information on the location of recurrence, which was recorded as local, regional or metastatic, and subsequent treatment and histopathological data.

### Follow-Up

All patients underwent clinical examination, as well contralateral mammography, annually for a minimum of 5 years before returning to the National Health Service (NHS) breast screening program.

Local recurrence was classified as ipsilateral skin, subcutaneous or chest wall recurrence, while contralateral recurrence was defined as contralateral breast parenchyma disease, both of which were proven by histopathological biopsy. Regional recurrence was classified as ipsilateral regional lymph node recurrence, either axillary, supraclavicular or internal mammary clearance, while metastatic recurrence was defined as any recurrence distant to the aforementioned sites. We included all patients who underwent a mastectomy for pure DCIS, and those individuals who had microinvasion or lobular carcinoma in situ (LCIS) were also included. All patients who had a definite invasive element and lymph involvement were excluded.

### Statistical Analysis

**Fig. 1:**
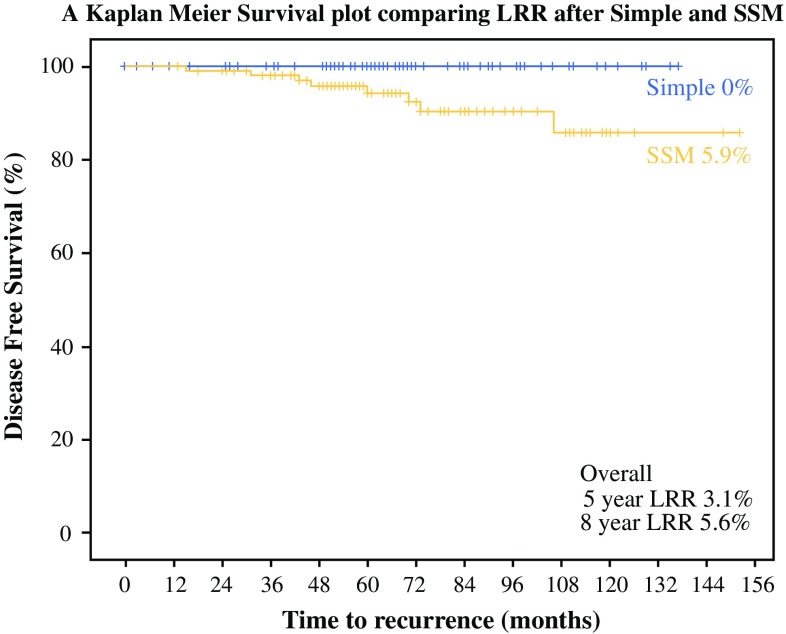
Kaplan–Meier curve comparing local recurrence after simple mastectomy and SSM. *SSM* skin-sparing mastectomy, *LRR* locoregional recurrence

The data were collected and analyzed using SPSS version 22.0 (IBM Corporation, Armonk, NY, USA). The Student’s *t* test was used to compare continuous variables between two groups, and the Chi-square test was used to compare categorical variables. Survival was evaluated using Kaplan–Meier survival curves and the log-rank test was used to compare survival between the two groups. As there were only eight recurrences, we had insufficient numbers for a robust regression analysis. The conventional 5% significance level was used (Fig. 1).

## Results

In total, 199 patients underwent a mastectomy for DCIS between January 2000 and December 2010, with a median follow-up time of 65 months (range 0–152).

SSM was undertaken on 102 patients and 97 had a simple mastectomy. Table 1 highlights the different demographic and histopathological features, as well as ER status, between the two groups.

**Table 1:** Characteristics of the simple mastectomy and SSM patient groups

	Simple (*n* = 97)	SSM (*n* = 102)	*p*-Value
Mean age, years (range)	61 (40–81)	53 (33–71)	<0.01^a^
Symptomatic presentation	28	36	0.72^b^
Size of DCIS, mm (range)	39 (2–130)	38 (1–125)	0.80^a^
High-grade	28	33	0.40^b^
Involved margin, <2 mm	26	31	0.38^b^
ER-negative	43	28	0.03^b^
HER2-positive	87	67	0.06^b^
Ipsilateral 5-year LRR	0	5.9	0.01^c^
Contralateral 5-year LRR	4.8	3.2	0.68^c^

Data are expressed as percentages unless otherwise specified

*SSM* skin-sparing mastectomy, *DCIS* ductal carcinoma in situ, *ER* estrogen receptor, *HER2* human epidermal growth factor receptor, *LRR* locoregional recurrence

^a^ Student’s *t* test

^b^ Chi-square test

^c^ Log-rank

Sixty-eight percent of patients presented with a screen-detected lesion and 32% presented symptomatically. SSM patients were younger, with a mean age of 53 years compared with 61 years in the simple mastectomy group (*p* *<* 0.01). No difference in the size of DCIS excised, the percentage of high-grade DCIS, or margin involvement was found between the SSM (31%) and simple mastectomy (26%) groups (Table 1). Patients undergoing simple mastectomy were more likely to be ER-negative, and 95.7% of ER-negative patients were HER2 positive.

### Type of Reconstruction

Of 102 patients treated by SSM, none were nipple-sparing mastectomies, 65 (63.7%) underwent immediate one-stage reconstruction, and 37 (36.3%) had insertion of a tissue expander followed by definitive reconstruction (Table 2).

**Table 2:** Reconstructive methods used on 102 patients undergoing SSM

Type of reconstruction	Number of patients	LRR
TE then implant	37 (36.3)	1 (2.7)
One stage	65 (63.7)	7 (10.8)
Implant	6 (5.9)	0
Autologous LD flap	49 (48)	4 (8.1)
Autologous TRAM flap	5 (4.9)	2 (40)
Autologous DIEP flap	5 (4.9)	1 (20)
Data are expressed as *n* (%)		

*SSM* skin-sparing mastectomy, *LRR* locoregional recurrence, *LD* latissimus dorsi, *TRAM* transverse rectus abdominis muscle, *DIEP* deep inferior epigastric perforator

### Pathology

LCIS was present in conjunction with DCIS in 13 patients, and definite or possible microinvasion was present in 19 patients.

### Recurrence

During the 10-year analysis period, eight LRRs were noted, all in the SSM group. There were no local recurrences after simple mastectomy. Kaplan–Meier analysis demonstrated that overall 5-year LRRs were 3.1% at 5 years and 5.6% at 8 years. LRR rates were higher in the SSM group, which had a 5.9% 5-year LRR compared with 0% in the simple mastectomy group (*p* *=* 0.012, log-rank).

Univariate analysis identified two factors that predicted risk of recurrence: a young age at mastectomy (<50 years of age) and close (<2 mm) or involved margins. Screen-detected LRR was 4.5% (6/132), similar to 3.4% (2/59) for symptomatic presentation. In general, high-grade and ER-negative tumors were more likely to recur, however there were insufficient events to confirm this.

### Contralateral Recurrence

The 5-year contralateral recurrence rate was 4.2%, rising to 8.5% at 8 years. Interestingly, the 5-year ipsilateral recurrence rate following SSM was higher at 5.9% than the 3.9% contralateral recurrence rate in the SSM group, suggesting that adequacy of excision played a key role.

### Analysis of Recurrence

All eight recurrences were IDC and presented as a lump either on clinical follow-up or symptomatically. Invasive recurrence represents a loss of local control and therefore potentially increases patient mortality. Median disease-free survival time was 55 months (range 15–106 months). Four of the eight recurrences had surrounding DCIS alongside the invasive component.

Of the eight recurrences, seven patients had immediate reconstruction at the time of their SSM. All eight of the recurrences had re-excision in the form of a WLE and axillary surgery (see Electronic Supplementary Table 1). Following recurrence, seven patients had adjuvant radiotherapy and seven had adjuvant chemotherapy (five with trastuzumab). Only three patients required endocrine treatment. One patient died after recurrent disease at 74 months post-surgery.

## Discussion

In this large UK series evaluating LRR after mastectomy for DCIS we found a 3.1% 5-year LRR, consistent with US results highlighting a higher LRR than the 1–2% historically quoted.^14^ LRR after SSM was 5.9% at 5 years compared with 0% after simple mastectomy. The increasing use of SSM may account for increasing LRR and is likely to be a consistent pattern with the use of SSM elsewhere. Previous papers demonstrated that young age at mastectomy (<50 years), as well as margin status, are important predictors of LRR.

A retrospective review of 223 patients with DCIS treated by SSM, with a mean follow-up of 82.3 months, reported a 5.1% LRR.^17^ The higher LRR, similar to our data, was associated with high-grade disease and close margins as predictors of LRR, with a 10.5% LRR in a <1 mm margin.^17^ In this series, six of the seven patients who recurred had residual breast tissue.^17^

After mastectomy, many surgeons in the UK are resistant to re-excision if margins, including the anterior (skin) margin, are involved. Our results highlight the importance of achieving clear margins and, if close, further surgical excision is required, even if this is overlying skin.

Fitzsullivan et al. reported 810 patients who underwent mastectomy for DCIS at the MD Anderson Cancer Center, with a median follow-up time of 75.6 months and demonstrating a 1% overall LRR, with a 5% LRR in those with a margin <1 mm.^19^ Their SSM group had an LRR of 1.5% (7/469), compared with 0.3% in the simple mastectomy group (1/341), a non-significant difference (*p* *=* 0.09).^19^ Intraoperative fresh frozen section analysis was routinely undertaken alongside radiographic imaging, leading to 14.3% (*n* = 116) of patients with a close margin of <3 mm, undergoing intraoperative re-excision, a practice not used in the UK.^19^ This re-excision led to a change in margin status from <3 mm to >3 mm in 103 patients (89%).^19^ Of the remaining 13 patients who had a margin of <3 mm following intraoperative re-excision, nine underwent a second operation for margin re-excision. This emphasis on achieving clear margins accounts for the lower 1% LRR and explains why LRR after SSM was not significantly higher despite resulting in a closer margin, as a higher proportion of SSM patients underwent intraoperative re-excision.^19^ Another study from the same institution highlighted the benefit of achieving clear margins for in situ and invasive disease in 1810 patients, demonstrating a 1% LRR when clear margins were achieved in 99.7% of patients.^20^ Increased rates of involved or close margins (29%) were found in patients undergoing SSM, as opposed to 12% in simple mastectomies in the study by Sheikh et al.; however, re-excision to achieve clear margins led to a low LRR at 28 months of 0.8% after simple mastectomy and 1.1% after SSM.^21^

Our unit had high rates of SSM use compared with the national average and this raises the question as to whether higher rates of SSM and immediate reconstruction have led to increased LRR. When undertaking SSM and immediate reconstruction, surgeons have to balance the further concern of pressure from the reconstruction on skin flaps and potential necrosis. Kim et al. reviewed recurrence excision specimens from 10 patients who developed LRR after SSM for DCIS. Five of the seven patients who underwent immediate reconstruction had residual breast tissue.^22^ The most commonly involved margin in all three series was the anterior margin.^17^,^19^,^23^

Post-mastectomy radiotherapy (PMRT) in patients who had a margin of <2 mm was recommended because of a 16% LRR in one study.^23^ In a survey of 226 surgeons in the UK, 19% said they would consider the use of PMRT, with 66% saying margin status was the key factor, but there is no evidence base for its use nor agreed margin width.^24^ Further work is required to evaluate differences in LRR between a margin of 1 and 2 mm to better inform risks of LRR with margin width and to counsel patients.

Half of the recurrence samples had DCIS associated with IDC, suggesting that DCIS had been left behind or that within residual tissue after mastectomy further DCIS had developed and the absence of radiological follow-up allowed invasive foci to supervene.

The median disease-free survival time in this study was 55 months, similar to previous reports^17^,^19^ and highlighting the importance of following up patients for at least 60 months. Invasive recurrence represents loss of local control and requires additional adjuvant treatment with chemotherapy, radiotherapy ± herceptin, which would not have been indicated for primary DCIS. Although only one patient died as a result of invasive recurrence, Bannani et al. found a 50% mortality rate when disease reoccurred.^25^

There is no accepted national surveillance policy for the ipsilateral chest wall or reconstructed breast following mastectomy for DCIS. The use of screening mammography when following up patients who have had an SSM with a transverse rectus abdominis muscle flap reconstruction has been shown to detect recurrences earlier while the disease is still in situ.^26^ However, few data are available to assess the effectiveness of mammography in patients undergoing SSM and immediate reconstruction.

Audits of LRR after mastectomy for DCIS have been large, retrospective, single-institution reviews; however, a large, prospective, multicenter audit of LRR after SSM is required. Repeated audit of post-mastectomy LRR has reduced LRR across all Dutch hospitals to less than 5% at 5 years, and a similar system of surgical quality control of LRR would likely reduce LRR in the UK.^27^

Univariate analysis demonstrated higher LRR in women under 40 years of age, a finding reported by others, with a 7.5% LRR <40 years of age as opposed to 1.5% >40 years of age.^28^ Bannani et al. also demonstrated that patients under 40 years of age had higher rates of LRR (14.2%) compared with 2.5% in patients over 40 years of age.^25^ Symptomatic DCIS and premenopausal women have a higher incidence of ER-negative disease that recurs earlier, usually within the first 3 years.^29^ Seven of the eight recurrences occurred in young women or ER-negative DCIS, and surgeons need to ensure clear margins and careful surveillance of these patients (Table 3).

**Table 3:** Analysis of univariate factors predicting risk of recurrence

	Recurrence (*n* = 8)	Non-recurrence (*n* = 191)	HR (95% CI); *p*-value
Mean age, years (range)	48 (37–54)	57 (33–81)	0.92 (0.85–0.99); 0.028
Involved margins, <2 mm	5	52	4.39 (1.02–17.94); 0.046
High-grade	8	131	39.10 (0.085–18130.86); 0.241
Size, mm (range)	48 (20–80)	38 (1–90)	1.01 (0.99–1.04); 0.414
Microinvasion	25.0%	9.6%	2.21 (0.94–5.20); 0.067
Comedonecrosis	28.6%	22.8%	2.12 (0.75–6.00); 0.155
ER-negative	5	57	3.14 (0.75–13.13); 0.118
HER2-positive	83.3%	16.7%	1.66 (0.19–14.48); 0.644
Symptomatic presentation	25%	35%	0.62 (0.122–3.10); 0.56

^1^Cox proportional hazard regression analysis

*HR* hazard ratio, *CI* confidence interval, *ER* estrogen receptor, *HER2* human epidermal growth factor receptor 2

## Conclusions

Surgeons must achieve clear margins and consider re-excision, including overlying skin, following SSM, particularly in young women with high-grade and ER-negative DCIS, in order to prevent LRR. Women undergoing mastectomy for DCIS should be counseled as to the potential increased need for further surgical excision which affects the final aesthetic outcome but lowers LRR. Further multicenter studies are necessary to evaluate LRR after SSM and the role of post-reconstruction mammography to aid earlier detection of LRR.

## Electronic supplementary material

Below is the link to the electronic supplementary material.

Supplementary material 1 (DOCX 14 kb)
