# Supplementary material for: Comparison of Local Recurrence After Simple and Skin-Sparing Mastectomy Performed in Patients with Ductal Carcinoma In Situ
Source: Ann Surg Oncol. 2016 Nov 11;24(4):1071–6. doi: 10.1245/s10434-016-5673-6 (PMC5339328; doi:10.1245/s10434-016-5673-6)
Supplement: Supplementary file 1 — Supplementary material 1 (DOCX 14 kb) [file 10434_2016_5673_MOESM1_ESM.docx]

Supplementary Table 1:

| Original DCIS pathology | Reconstructive strategy | Time to recurrence (months) | Recurrence  pathology | Recurrence surgery | Recurrence adjuvant treatment |
| --- | --- | --- | --- | --- | --- |
| 39yo High/48mm - 7/8/na >2mm | Immediate  TRAM | 60 | IDC/III/16mm 8/7/3+  0 of 2 LN | WLE + SLNB | RT + CT + Herceptin + ET |
| 54yo - High/40mm- 0/0/3+ <1mm | Immediate  LD + implant | 43 | IDC/III/25mm 0/0/3+  0 of 1 LN | WLE + SLNB | RT + CT + Herceptin |
| 57yo - High/45mm - 0/0/0 <1mm | Delayed, TE then implant | 73 | IDC/II/4mm -0/0/0  0 of 4 LN | WLE + ANS | RT |
| 52yo - High/70mm/multi - 8/7/2- <2mm | Immediate LD + implant | 46 | IDC/III/48mm - 8/7/na  0 of 3 LN | WLE + SLNB  + further WLE | CT + ET |
| 51yo - High/48mm - 0/0/3+ <2mm | Immediate LD + implant | 31 | IDC/III/30mm - 0/0/3+  0 of 25 LN | WLE +ANC | RT + CT + Herceptin |
| 37yo - High/35mm - 8/4/na >2mm | Immediate LD + implant | 106 | IDC/III/20mm 8/5/na  0 of 4 LN | WLE+ SLNB | RT + CT + ET |
| 48 yo – High/ 51mm- 0/0/3+ <1mm | Immediate DIEP | 15 | IDC/III/12mm 0/0/3+  0 of 2 LN | WLE + ANS | RT + CT + Herceptin |
| 50 yo - High/20mm - 0/0/3+ >2mm | Immediate TRAM | 70 | IDC/III/55mm 0/0/3+  9 of 9 LN | WLE + ANC | RT, CT + Herceptin |
